# Associations of Alzheimer's-related plasma biomarkers with cognitive decline in Parkinson's disease

**DOI:** 10.1007/s00415-023-11875-z

**Published:** 2023-07-22

**Authors:** Yasuaki Mizutani, Reiko Ohdake, Harutsugu Tatebe, Atsuhiro Higashi, Sayuri Shima, Akihiro Ueda, Mizuki Ito, Takahiko Tokuda, Hirohisa Watanabe

**Affiliations:** 1 Department of Neurology, Fujita Health University School of Medicine, 1-98 Dengakugakugo, Kutsukake-Cho, Toyoake, Aichi 470-1192 Japan; 2 Department of Functional Brain Imaging, Institute for Quantum Medical Science, National Institutes for Quantum Science and Technology, Chiba, Chiba Japan

**Keywords:** Parkinson’s disease (PD), Alzheimer’s disease (AD), Cognitive impairment, Plasma biomarker

## Abstract

**Background:**

Parkinson’s disease (PD) is associated with cognitive decline through multiple mechanisms, including Alzheimer’s disease (AD) pathology and cortical Lewy body involvement. However, its underlying mechanisms remain unclear. Recently, AD-related plasma biomarkers have emerged as potential tools for predicting abnormal pathological protein accumulation. We aimed to investigate the association between AD-related plasma biomarkers and cognitive decline in PD patients.

**Methods:**

Plasma biomarkers were measured in 70 PD patients (49 with nondemented Parkinson’s disease (PDND) and 21 with Parkinson’s disease dementia (PDD)) and 38 healthy controls (HCs) using a single-molecule array. The study evaluated (1) the correlation between plasma biomarkers and clinical parameters, (2) receiver operating characteristic curves and areas under the curve to evaluate the discrimination capacity of plasma biomarkers among groups, and (3) a generalized linear model to analyze associations with Addenbrooke’s Cognitive Examination-Revised and Montreal Cognitive Assessment-Japanese version scores.

**Results:**

Plasma glial fibrillary acidic protein significantly correlated with cognitive function tests, including all subdomains, with a notable increase in the PDD group compared with the HC and PDND groups, while plasma neurofilament light chain captured both cognitive decline and disease severity in the PDND and PDD groups. Plasma beta-amyloid 42/40 and pholphorylated-tau181 indicated AD pathology in the PDD group, but plasma beta-amyloid 42/40 was increased in the PDND group compared with HCs and decreased in the PDD group compared with the PDND group.

**Conclusions:**

AD-related plasma biomarkers may predict cognitive decline in PD and uncover underlying mechanisms suggesting astrocytic pathologies related to cognitive decline in PD.

**Supplementary Information:**

The online version contains supplementary material available at 10.1007/s00415-023-11875-z.

## Introduction

Parkinson’s disease (PD) is the second most prevalent neurodegenerative disorder, after Alzheimer’s disease (AD). The prevalence of PD increases with age, and the number of patients is increasing proportionally with the aging population [1]. Although numerous symptomatic treatments have been developed for motor symptoms, cognitive impairment remains a significant challenge for patients with PD and their caregivers, as it is closely associated with a poor quality of life (QOL) and caregiver burden [2]. A recent systematic review and meta-analysis demonstrated that 26.3% of patients with PD were diagnosed with dementia (PDD) [3]; however, the underlying mechanisms of cognitive decline in PD are not entirely understood, and multiple mechanisms have been reported, including AD pathologies and cortical involvement of Lewy bodies [4–6].

The recent advances in blood-based biomarkers of pathological changes in AD, such as plasma beta-amyloid (Aβ) and phosphorylated tau (p-tau), have made these biomarkers more accessible and their evaluation more prevalent [7, 8]. Plasma Aβ42/40 is a reliable marker of the neocortical Aβ burden, as validated by positron emission tomography (PET) in AD patients [9], whereas plasma p-tau181 correlates with Aβ and tau PET uptake [10]. In addition, plasma neurofilament light chain (NfL), a marker of axonal damage, increases in various neurodegenerative diseases [11], and plasma glial fibrillary acidic protein (GFAP) is a marker of reactive astrogliosis and is elevated in the early stages of AD [12]. The recent studies have demonstrated an association between AD-related plasma biomarkers and cognitive impairment in patients with PD [13, 14]. However, the biomarkers analyzed in these studies were limited, and a comprehensive comparison of the clinical significance of multiple biomarkers has not been performed.

Cognitive impairment in PD is believed to be heterogeneous and span multiple cognitive domains, such as memory, attention, visuospatial abilities, and executive functions [6, 15, 16]. Moreover, the cognitive function domains that affect QOL can differ with the progression of cognitive dysfunction in patients with PD [17]. Therefore, this study aimed to compare six plasma biomarkers (Aβ42, Aβ40, Aβ42/40, p-tau181, GFAP, and NfL) between patients with PD and healthy controls (HCs) and investigate their association with comprehensive clinical parameters, including subitems of cognitive scales, to provide insights into the link between AD-related plasma biomarkers and the clinical presentation of cognitive impairment in PD.

## Methods

### Participants

We recruited 70 consecutive patients with PD admitted to Fujita Health University Hospital between May 2020 and September 2021. All patients with PD fulfilled the Movement Disorder Society (MDS) clinical diagnostic criteria [18]. Patients with PD were further subcategorized into nondemented Parkinson’s disease (PDND) and Parkinson’s disease dementia (PDD) groups [19], with the latter diagnosed based on the MDS Task Force algorithm [20]. In the PD group, 21 patients (30.0%) were diagnosed with PDD, while the remaining 49 patients were classified as having PDND. We also enrolled 38 age- and sex-matched HCs from our ongoing aging cohort study at Fujita Health University, Japan. HCs were included based on the following criteria: (1) cognitively normal with Mini-Mental State Examination (MMSE) scores greater than 25 [21] and an Addenbrooke’s Cognitive Examination-Revised (ACE-R) total score greater than 88 [22] without a history of neurological or psychiatric disorders and (2) no observable anatomical abnormality in the brain according to magnetic resonance imaging.

This study was approved by the ethics committee of Fujita Health University Hospital, and all participants provided written informed consent before participation, as well as opt-out consent.

### Clinical evaluation

Motor and nonmotor symptoms related to PD were assessed using the Japanese version of the Movement Disorder Society’s Unified PD Rating Scale (MDS-UPDRS) [23]. Cognitive performance was evaluated using the Frontal Assessment Battery (FAB) [24], ACE-R, MMSE, and the Japanese version of the Montreal Cognitive Assessment (MoCA-J) [25]. The MoCA was evaluated using the total score and six subscores consisting of attention, orientation, executive, memory, language, and visuospatial domains, which were proposed in the original MoCA manuscript [25]. The ACE-R was evaluated using the total score and five subscores comprising orientation and attention, memory, verbal fluency, language, and visuospatial abilities. We also assessed the participants using the Parkinson’s Disease Questionnaire-39 Summary Index, Geriatric Depression Scale-15, Odor Stick Identification Test for the Japanese (OSIT-J) score, Japanese version of the REM Sleep Behavior Disorder Screening Questionnaire, Scales for Outcomes in Parkinson’s Disease-Autonomic, Epworth Sleepiness Scale, and Japanese version of the Questionnaire for Impulsive − Compulsive Disorders in Parkinson’s Disease. The levodopa equivalent daily dose was calculated according to established formulae [26] with additional consideration of opicapone and safinamide intake [27]. We performed all neurological evaluations of patients with PD during the ‘on’ condition.

### Sample collection and assays of plasma biomarkers

To obtain plasma from all recruited participants, blood samples were collected after more than 6 h of fasting. The samples were centrifuged for 10 min at 1500*g*, and 500 µL aliquots of plasma were immediately frozen and stored at − 80 °C until assayed. Each aliquot was divided to avoid repeated freezing and thawing. The plasma GFAP, NfL, Aβ40, Aβ42, and p-tau181 levels were determined with a single-molecule array (Simoa) using the Simoa Human Neurology 4-Plex E kit and the Simoa pTau-181 V2 Advantage kit (Quanterix, Billerica, MA, USA), according to the manufacturer’s protocol. Plasma samples were tested in duplicate. In the analysis of plasma NfL levels, we excluded one patient with PD who had a recent traumatic episode.

### Statistical analysis

JMP software, version 16 (SAS Institute, Cary, NC, USA) was used for statistical analyses. Differences were considered statistically significant at a value of *p* < 0.05. Fisher’s exact test was used to compare the sex distribution between the two groups. We assessed the normality of the variables and homoscedasticity using the Shapiro–Wilk test and Levene’s test, respectively. The Wilcoxon’s rank sum test was used to compare continuous variables between the two groups because assumptions of normality or homogeneity of variance were violated. Statistical significance among the three groups was analyzed using the Kruskal–Wallis test followed by post hoc Steel–Dwass multiple comparison tests. Correlations between continuous variables were assessed using Spearman’s rank correlation test. Continuous variables are expressed as the mean ± standard deviation. In addition, we used a generalized linear model to evaluate the effects of plasma biomarkers and clinical parameters on the MoCA-J and ACE-R total scores, based on the outcomes of the univariate analyses. To evaluate the discrimination capacity of plasma biomarkers among groups, a receiver-operating characteristic (ROC) curve was constructed and the area under the curve (AUC) was determined.

## Results

### Participant characteristics

Table 1 illustrates the clinical characteristics of individuals in the PD and HC groups. Patients with PD exhibited significantly lower education levels (*p* = 0.0146), higher depression scores (Geriatric Depression Scale-15: *p* < 0.0001), and lower scores on global cognitive scales (MMSE, ACE-R total score, and MoCA-J total score: *p* < 0.0001) than did HCs but did not differ in age at examination and sex. Supplementary Table 1 summarizes the clinical characteristics of the individuals in the HC, PDND, and PDD groups. Notably, patients with PDD displayed a significantly older age at examination (*p* = 0.0104) and onset (*p* = 0.0127), a lower education level (*p* = 0.0121), a higher Hoehn and Yahr (HY) scale (*p* = 0.0372), more severe hyposmia (OSIT-J score: *p* = 0.0003), and lower scores on global cognitive scales (MMSE, ACE-R total score, and MoCA-J total score: *p* < 0.0001) than did patients with PDND.

**Table 1:** The clinical characteristics of the participants in the PD and HC group

Characteristics	HC (*N* = 38) Mean ± SD (range)	PD (*N* = 70) Mean ± SD (range)	*p* value
Age at examination (years)	68.4 ± 4.89 (61–79)	69.7 ± 8.40 (48–82)	0.1032
Sex (male/female)	19/19	41/29	0.4229
Age at onset (years)		62.1 ± 9.78 (36–77)	
Disease duration (months)		91.3 ± 50.6 (9–237)	
Education (years)	13.9 ± 1.80 (12–16)	12.7 ± 2.58 (9–18)	**0.0146**
LEDD (mg)		647 ± 339 (0–1425)	
MDS-UPDRS I		10.8 ± 5.83 (0–25)	
MDS-UPDRS II		15.1 ± 8.34 (0–36)	
MDS-UPDRS III		35.6 ± 16.4 (7–78)	
MDS-UPDRS IV		5.41 ± 4.47 (0–14)	
HY		3.11 ± 1.04 (1–5)	
PDQ-39 SI		30.1 ± 15.8 (2.1–71.3)	
SCOPA-AUTO		15.0 ± 8.03 (2–47)	
GDS-15	2.55 ± 2.16 (0–8)	6.91 ± 3.73 (0–15)	** < 0.0001**
J-QUIP		0.657 ± 0.976 (0–3)	
RBDSQ-J		5.24 ± 2.95 (1–11)	
ESS		9.20 ± 5.87 (0–24)	
OSIT-J score		3.59 ± 2.54 (0–11)	
MMSE	28.7 ± 1.19 (26–30)	26.0 ± 4.35 (5–30)	** < 0.0001**
ACE-R total score	95.0 ± 2.99 (89–100)	83.0 ± 15.4 (17–99)	** < 0.0001**
MoCA-J total score	24.8 ± 2.67 (20–30)	21.1 ± 5.07 (2–29)	** < 0.0001**
FAB		13.0 ± 2.80 (5–18)	

Significance was tested using the Wilcoxon rank sum test

*PD* Parkinson's disease, *HC* healthy control, *LEDD* Levodopa equivalent daily dose, *MDS-UPDRS* Movement Disorder Society’s Unified Parkinson's Disease Rating Scale, *HY* Hoehn − Yahr scale, *PDQ-39 SI* Parkinson's Disease Questionnaire-39 Summary Index, *SCOPA-AUT* Scales for Outcomes in Parkinson's Disease-Autonomic, *GDS-15* Geriatric Depression Scale-15, *J-QUIP* Japanese version of the Questionnaire for Impulsive − Compulsive Disorders in Parkinson's Disease, *RBDSQ-J* Japanese version of the REM Sleep Behavior Disorder Screening Questionnaire, *ESS* Epworth Sleepiness Scale, *OSIT-J* Odor Stick Identification Test for Japanese, *MMSE* Mini-Mental State Examination, *ACE-R* Addenbrooke’s Cognitive Examination-Revised, *MoCA-J* Japanese version of the Montreal Cognitive Assessment, *FAB* Frontal Assessment Battery

*Bold letters indicate a statistically significant difference

### Comparison of plasma biomarker levels between the PD and HC groups

**Fig. 1:**
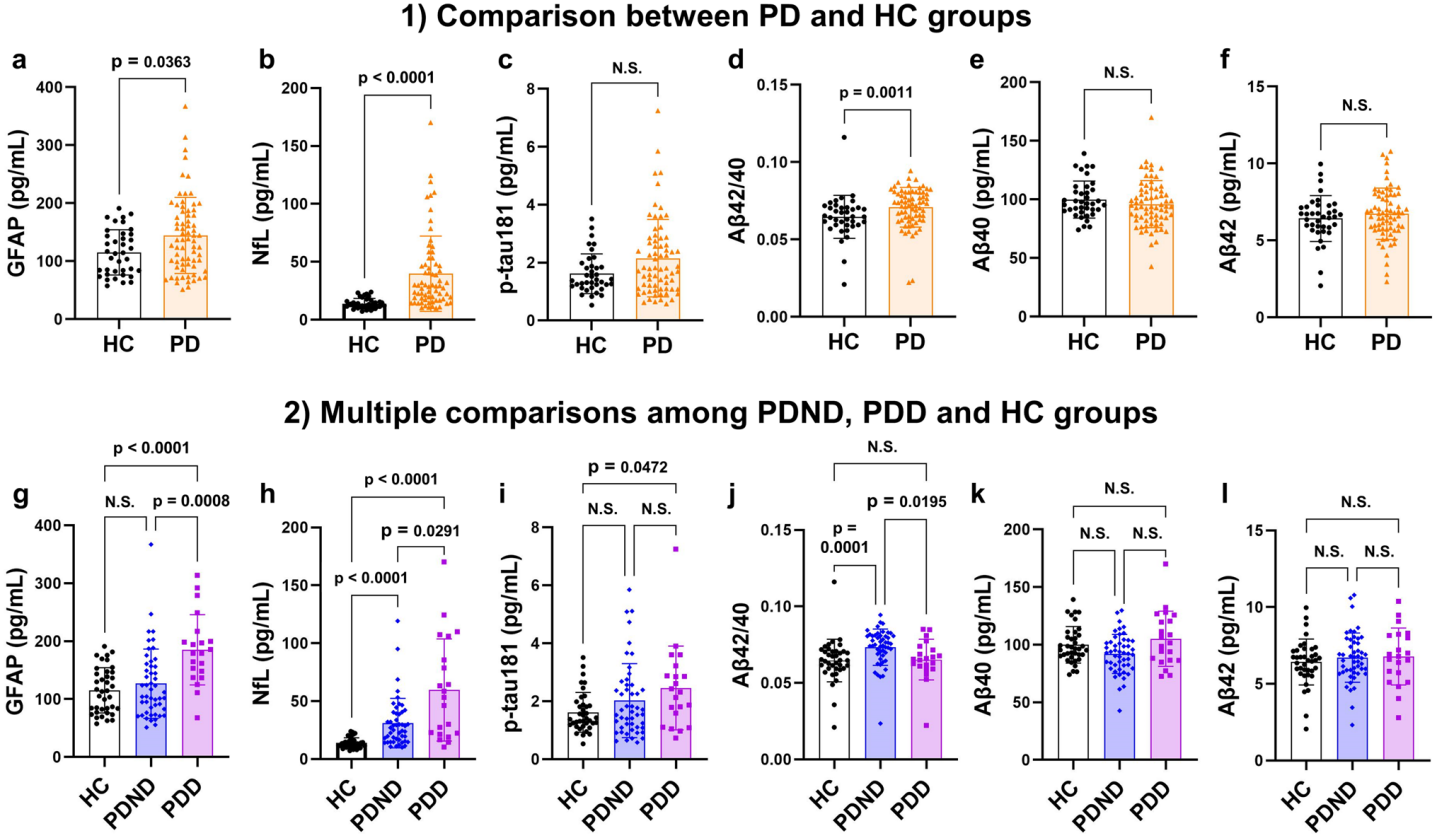
Comparison of plasma biomarker levels. **1** Comparison results between the two groups of PD and HC in plasma levels of GFAP (**a**), NfL (**b**), p-tau181 (**c**), Aβ42/40 (**d**), Aβ40 (**e**) and Aβ42 (**f**). Significance was tested using the Wilcoxon rank sum test. **2** Multiple comparison results among the three groups of HC, PDND, and PDD in plasma levels of GFAP (**g**), NfL (**h**), p-tau181 (**i**), Aβ42/40 (**j**), Aβ40 (**k**) and Aβ42 (**l**). Significance was tested with Kruskal − Wallis test followed by post hoc Steel − Dwass multiple comparison tests. *PD* Parkinson’s disease, *HC* healthy control, *PDND* nondemented Parkinson’s disease, *PDD* Parkinson’s disease dementia, *GFAP* glial fibrillary acidic protein, *NfL* neurofilament light chain, *p-tau* phosphorylated tau, *Aβ* amyloid beta, *N.S.* not significant

Figure 1a and b demonstrates that the GFAP and NfL levels were significantly higher in the PD group than in the HC group (PD group: 144 ± 65.6 pg/mL, HC group: 115 ± 39.1 pg/mL, *p* = 0.0363; and PD group: 39.8 ± 32.5 pg/mL, HC group: 13.9 ± 4.45 pg/mL, *p* < 0.0001; respectively). Conversely, there was no significant difference in the p-tau181 level between the two groups (PD group: 2.15 ± 1.33 pg/mL, HC group: 1.62 ± 0.684 pg/mL, *p* = 0.0989) (Fig. 1c). With regard to Aβ, patients with PD exhibited significantly higher Aβ42/40 levels than did HCs (PD group: 0.0709 ± 0.0128, HC group: 0.0646 ± 0.0139, *p* = 0.0011) (Fig. 1d), whereas the analyses of Aβ40 and Aβ42 separately showed no significant difference between the PD and HC groups (PD group: 96.0 ± 20.0 pg/mL, HC group: 100.0 ± 15.8 pg/mL, *p* = 0.2429; and PD group: 6.74 ± 1.67 pg/mL, HC group: 6.43 ± 1.49 nmol/h/mL, *p* = 0.3940; respectively) (Fig. 1e, f).

### Association of plasma biomarkers with clinical parameters in PD patients

**Fig. 2:**
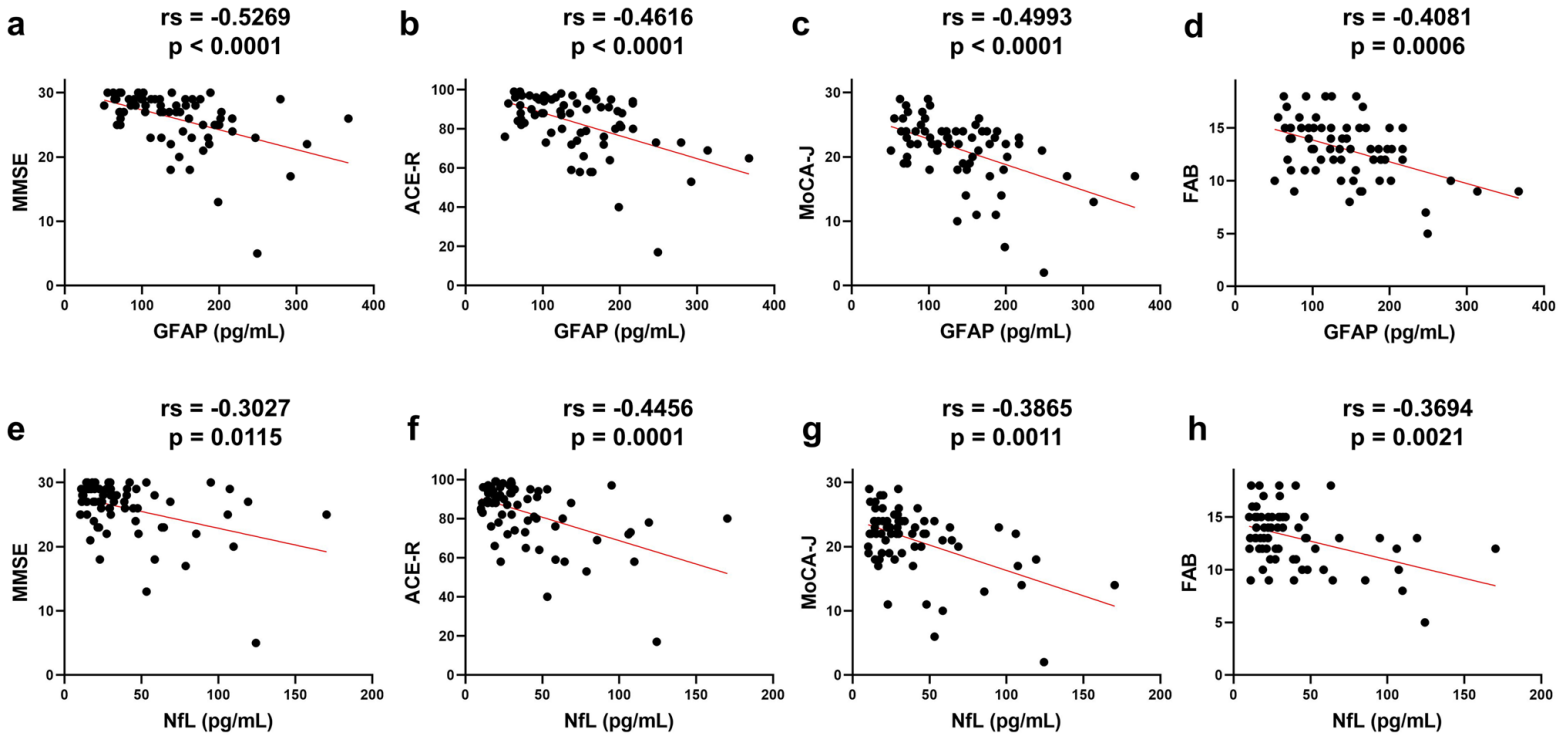
Correlation between the levels of plasma GFAP or NfL and the scores of cognitive scales in the PD group. Correlation between plasma GFAP levels and MMSE (**a**), ACE-R (**b**), MoCA-J (**c**), and FAB (**d**). Correlation between plasma NfL levels and MMSE (**e**), ACE-R (**f**), MoCA-J (**g**), and FAB (**h**). The Spearman’s rank correlation test was used to determine significant correlations. *PD* Parkinson’s disease, *GFAP* glial fibrillary acidic protein, *NfL* neurofilament light chain, *MMSE* Mini-Mental State Examination, *ACE-R* Addenbrooke’s Cognitive Examination-Revised, *MoCA-J* Japanese version of the Montreal Cognitive Assessment, *FAB* Frontal Assessment Battery

Table 2 summarizes the relationships between the six plasma biomarkers and clinical indices in the PD group. Plasma GFAP, NfL, p-tau181, and Aβ40 levels were significantly positively correlated with age at examination and age at onset. Significant positive correlations were found between GFAP and MDS-UPDRS I scores, NfL and MDS-UPDRS II scores, and GFAP, NfL, p-tau181, or Aβ40 and the HY scale. GFAP also showed a significant negative correlation with OSIT-J scores. Furthermore, NfL and especially GFAP were significantly negatively correlated with MMSE, ACE-R, MoCA-J, and FAB scores (Fig. 2a–h). However, Aβ40 was weakly correlated with MMSE and ACE-R scores, Aβ42/40 was only weakly correlated with MMSE scores, and p-tau181 was weakly correlated with ACE-R and FAB scores. Notably, GFAP was negatively correlated with all ACE-R and MoCA-J subscores; NfL was negatively correlated with all but the language subscore of the MoCA-J, while p-tau181, Aβ42/40, Aβ40, and Aβ42 showed significant correlations with only some subscores (Table 3). Conversely, in the HC group, these six plasma biomarkers showed no significant correlation with MMSE, ACE-R, or MoCA-J scores.

**Table 2:** The correlations between plasma biomarkers and clinical parameters in the PD group

	GFAP		NfL		p-tau181		Aβ42/40		Aβ40		Aβ42	
	rs	*p* value	rs	*p* value	rs	*p* value	rs	*p* value	rs	*p* value	rs	*p* value
Age at examination	**0.5479**	** < 0.0001**	**0.5552**	** < 0.0001**	**0.4505**	< 0.0001	− 0.2206	0.0664	**0.4042**	**0.0005**	0.2123	0.0777
Sex		0.1525		0.2287		0.6082		0.5834		0.5197		0.8394
Age at onset	**0.4023**	**0.0006**	**0.3816**	**0.0012**	**0.3802**	**0.0012**	− 0.1853	0.1246	**0.2738**	**0.0218**	0.1066	0.3799
Disease duration	0.0444	0.7149	0.1310	0.2833	− 0.0042	0.9722	0.0127	0.9166	0.1100	0.3646	0.1400	0.2476
Education	− 0.1837	0.1278	− 0.2194	0.0701	0.0632	0.6035	0.1069	0.3783	− 0.1409	0.2448	− 0.0951	0.4337
LEDD	0.0896	0.4605	0.2201	0.0691	0.0923	0.4474	− 0.1220	0.3145	0.1051	0.3867	0.2202	0.0670
MDS-UPDRS I	**0.2676**	**0.0262**	0.1595	0.1938	0.1832	0.1319	− 0.0633	0.6055	0.1214	0.3206	− 0.0158	0.8975
MDS-UPDRS II	0.0134	0.9131	**0.3071**	**0.0109**	0.0704	0.5654	− 0.0029	0.9812	0.1393	0.2538	0.0463	0.7056
MDS-UPDRS III	0.0325	0.7940	0.1627	0.1919	0.1695	0.1703	− 0.0885	0.4763	0.0920	0.4589	− 0.0149	0.9048
MDS-UPDRS IV	0.0308	0.8063	0.1395	0.2676	− 0.1217	0.3303	− 0.0942	0.4519	0.2027	0.1027	0.1187	0.3425
HY	**0.3305**	**0.0052**	**0.4941**	** < 0.0001**	**0.4245**	**0.0002**	− 0.1864	0.1222	**0.2572**	**0.0316**	0.0860	0.4792
PDQ-39 SI	0.0765	0.5292	0.1150	0.3467	0.1341	0.2682	− 0.0210	0.8627	0.0968	0.4251	0.0169	0.8893
SCOPA-AUTO	0.0179	0.8832	− 0.0639	0.6018	− 0.0928	0.4450	0.0857	0.4803	− 0.0542	0.6556	0.0182	0.8811
GDS-15	0.1813	0.1331	0.1631	0.1807	− 0.0063	0.9587	0.0447	0.7135	0.0337	0.7818	0.0779	0.5217
J-QUIP	0.1016	0.4027	0.0442	0.7185	0.1395	0.2493	0.2160	0.0725	0.0633	0.6025	0.1888	0.1175
RBDSQ-J	0.0883	0.4671	0.1551	0.2033	0.1679	0.1646	− 0.1512	0.2116	0.1656	0.1706	0.0717	0.5552
ESS	− 0.0363	0.7657	0.0290	0.8129	0.0530	0.6628	0.0369	0.7619	0.1339	0.2693	0.1236	0.3081
OSIT-J score	**− 0.2725**	**0.0235**	− 0.2064	0.0913	− 0.2340	0.0530	0.0439	0.7205	− 0.0367	0.7648	− 0.0919	0.4524
MMSE	**− 0.5269**	** < 0.0001**	**− 0.3027**	**0.0115**	− 0.1655	0.1711	**0.2837**	**0.0173**	**− 0.2494**	**0.0373**	− 0.1205	0.3202
ACE-R total score	**− 0.4616**	** < 0.0001**	**− 0.4456**	**0.0001**	**− 0.2507**	**0.0363**	0.1500	0.2151	**− 0.3039**	**0.0105**	− 0.1910	0.1132
MoCA-J total score	**− 0.4993**	** < 0.0001**	**− 0.3865**	**0.0011**	− 0.2243	0.0640	0.1792	0.1406	− 0.1699	0.1627	− 0.0597	0.6261
FAB	**− 0.4081**	**0.0006**	**− 0.3694**	**0.0021**	**− 0.2702**	**0.0259**	0.1194	0.3320	− 0.1194	0.3320	− 0.0581	0.6382

Spearman’s rank correlation test was used to determine significant correlations

*GFAP* glial fibrillary acidic protein, *NfL* neurofilament light chain, *p-tau* phosphorylated tau, *Aβ* amyloid beta, *PD* Parkinson's disease, *LEDD* Levodopa equivalent daily dose, *MDS-UPDRS* Movement Disorder Society’s Unified Parkinson's disease Rating Scale, *HY* Hoehn − Yahr scale, *PDQ-39 SI* Parkinson's Disease Questionnaire-39 Summary Index, *SCOPA-AUT* Scales for Outcomes in Parkinson's Disease-Autonomic, *GDS-15* Geriatric Depression Scale-15, *J-QUIP* Japanese version of the Questionnaire for Impulsive − Compulsive Disorders in Parkinson's Disease, *RBDSQ-J* Japanese version of the REM Sleep Behavior Disorder Screening Questionnaire, *ESS* Epworth Sleepiness Scale, *OSIT-J* Odor Stick Identification Test for Japanese, *MMSE* Mini-Mental State Examination, *ACE-R* Addenbrooke’s Cognitive Examination-Revised, *MoCA-J* Japanese version of the Montreal Cognitive Assessment, *FAB* Frontal Assessment Battery

*Bold letters indicate a correlation with a statistically significant difference

**Underlines indicate a moderate or higher correlation coefficient (absolute value of rs > 0.4)

**Table 3:** The correlations between plasma biomarkers and subscores of ACE-R or MoCA-J in the PD group

	GFAP		NfL		p-tau181		Aβ42/40		Aβ40		Aβ42	
	rs	*p* value	rs	*p* value	rs	*p* value	rs	*p* value	rs	*p* value	rs	*p* value
ACE-R												
Attention/orientation	**− 0.5515**	** < 0.0001**	**− 0.3824**	**0.0012**	**− 0.2535**	**0.0342**	0.2165	0.0718	− 0.2000	0.0969	− 0.1139	0.3476
Memory	**− 0.3971**	**0.0007**	**− 0.3048**	**0.0109**	− 0.1674	0.1659	0.1348	0.2658	− 0.2043	0.0898	− 0.1265	0.2968
Fluency	**− 0.3494**	**0.0030**	**− 0.4815**	** < 0.0001**	**− 0.2674**	**0.0253**	0.1717	0.1552	**− 0.3159**	**0.0077**	− 0.1550	0.2000
Language	**− 0.2983**	**0.0121**	**− 0.2765**	**0.0214**	− 0.0211	0.8621	0.0405	0.7391	− 0.1426	0.2389	− 0.1528	0.2068
Visuospatial	**− 0.5041**	** < 0.0001**	**− 0.6111**	** < 0.0001**	**− 0.4097**	**0.0004**	**0.2353**	**0.0499**	**− 0.4047**	**0.0005**	− 0.1915	0.1122
MoCA-J												
Attention	**− 0.4118**	**0.0004**	**− 0.2474**	**0.0404**	− 0.0948	0.4350	**0.2439**	**0.0419**	− 0.1555	0.1987	− 0.0193	0.8741
Orientation	**− 0.4591**	** < 0.0001**	**− 0.4003**	**0.0007**	**− 0.3021**	**0.0116**	**0.3644**	**0.0021**	− 0.2240	0.0642	0.0115	0.9255
Executive function	**− 0.2577**	**0.0313**	**− 0.3423**	**0.0040**	− 0.0404	0.7397	0.2182	0.0695	− 0.1176	0.3323	0.0106	0.9304
Memory	**− 0.4221**	**0.0003**	**− 0.2561**	**0.0350**	− 0.2078	0.0866	0.0305	0.8034	− 0.0407	0.7401	− 0.0526	0.6680
Language	**− 0.2390**	**0.0463**	− 0.2269	0.0608	− 0.0705	0.5618	0.1069	0.3786	− 0.1037	0.3931	− 0.0149	0.9025
Visuospatial function	**− 0.3142**	**0.0081**	**− 0.3723**	**0.0016**	− 0.1492	0.2176	0.0835	0.4918	− 0.1189	0.3267	− 0.0212	0.8616

Spearman’s rank correlation test was used to determine significant correlations

*GFAP* glial fibrillary acidic protein, *NfL* neurofilament light chain, *p-tau* phosphorylated tau, *Aβ* amyloid beta, *PD* Parkinson's disease, *ACE-R* Addenbrooke’s Cognitive Examination-Revised, *MoCA-J* Japanese version of the Montreal Cognitive Assessment

*Bold letters indicate a correlation with a statistically significant difference

**Underlines indicate a moderate or higher correlation coefficient (absolute value of rs > 0.4)

### Comparison of plasma biomarker levels in the PDND, PDD, and HC groups

**Fig. 3:**
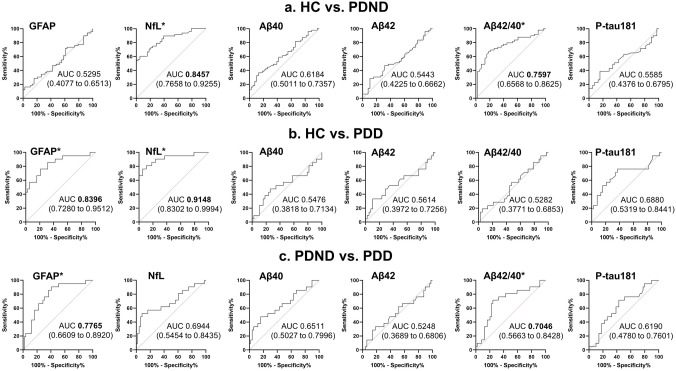
ROC analysis in the discrimination capacity of the six plasma biomarkers among groups. ROC curves and AUC for differentiation between HC and PDND (**a**), HC and PDD (**b**), PDD and PDND (**c**). The numbers in the brackets indicate the 95% confidence intervals for the AUC. *ROC* receiver operating characteristic, *AUC* area under the curve, *GFAP* glial fibrillary acidic protein, *NfL* neurofilament light chain, *Aβ* amyloid beta, *p-tau* phosphorylated tau, *HC* healthy control, *PDND* nondemented Parkinson’s disease, *PDD* Parkinson’s disease dementia. The asterisk denotes a biomarker with an acceptable or higher AUC value (> 0.7, bold letters)

Figure 1g–l and Supplementary Table 1 show the multiple comparison results of the six plasma biomarkers among the HC, PDND, and PDD groups. Steel–Dwass post hoc comparisons showed that patients with PDD had higher GFAP levels than did HCs (*p* < 0.0001) and patients with PDND (*p* = 0.0008) (Fig. 1g). The NfL levels significantly differentiated PDD patients from patients with PDND (*p* = 0.0291) and differentiated both patients with PDND (*p* < 0.0001) and those with PDD (*p* < 0.0001) from HCs (Fig. 1h). Regarding p-tau181 levels, there was a significant difference between HCs and patients with PDD but not between HCs and patients with PDND (Fig. 1i). Although no significant differences were observed in multiple comparisons of Aβ40 and Aβ42 (Fig. 1k, l), patients with PDND presented significantly higher Aβ42/40 levels than those of HCs (*p* = 0.0001) and PDD patients (*p* = 0.0195) (Fig. 1j). There were no significant differences between patients with PDD and HCs (Fig. 1j). Regarding the ROC analysis conducted on the six plasma biomarkers across various groups (Fig. 3 and Table 4), NfL and Aβ42/40 effectively distinguished the HC and PDND groups (AUC values: NfL, 0.8457; Aβ42/40, 0.7597), whereas GFAP and NfL exhibited excellent discriminatory potential in distinguishing the HC and PDD groups (AUC values: GFAP, 0.8396; NfL, 0.9148; GFAP*NfL, 0.9236). Notably, GFAP and Aβ42/40 demonstrated significant discriminatory capacity in differentiating between the PDND and PDD groups (AUC values: GFAP, 0.7765; Aβ42/40, 0.7046).

**Table 4:** The ROC analysis of the six plasma biomarkers in the discrimination capacity among the groups

Plasma biomarker	Cutoff value	Sensitivity; %	Specificity; %	AUC (95% CI)	*p* value
HC VS PDND					
GFAP	187.0	16.33	97.37	0.5295 (0.4077–0.6513)	0.6379
NfL	22.54	60.42	94.74	**0.8457** (0.7658–0.9255)	< 0.0001
Aβ40	85.28	36.73	86.84	0.6184 (0.5011–0.7357)	0.0592
Aβ42	7.644	28.57	89.47	0.5443 (0.4225–0.6662)	0.4802
Aβ42/40	0.07199	67.35	81.58	**0.7597** (0.6568–0.8625)	< 0.0001
p-tau181	2.244	38.78	84.21	0.5585 (0.4376–0.6795)	0.3509
HC VS PDD					
GFAP	148.0	76.19	78.95	**0.8396** (0.7280 to 0.9512)	< 0.0001
NfL	22.37	76.19	94.74	**0.9148** (0.8302 to 0.9994)	< 0.0001
Aβ40	107.3	47.62	73.68	0.5476 (0.3818 to 0.7134)	0.5475
Aβ42	7.985	33.33	89.47	0.5614 (0.3972 to 0.7256)	0.4379
Aβ42/40	0.07751	19.05	94.74	0.5282 (0.3771 to 0.6853)	0.7217
p-tau181	1.595	76.19	63.16	0.6880 (0.5319 to 0.8441)	0.0176
PDD VS PDND					
GFAP	125.1	90.48	59.18	**0.7765** (0.6609–0.8920)	0.0003
NfL	53.28	52.38	91.67	0.6944 (0.5454–0.8435)	0.0106
Aβ40	107.3	47.62	83.67	0.6511 (0.5027–0.7996)	0.0463
Aβ42	7.985	33.33	81.63	0.5248 (0.3689–0.6806)	0.7438
Aβ42/40	0.06884	71.43	75.51	**0.7046** (0.5663–0.8428)	0.0070
p-tau181	1.789	71.43	57.14	0.6190 (0.4780–0.7601)	0.1164

*ROC* receiver operating characteristic, *HC* healthy control, *PDND* nondemented Parkinson’s disease, *AUC* area under the curve, *CI* confidence intervals, *GFAP* glial fibrillary acidic protein, *NfL* neurofilament light chain, *Aβ* amyloid beta, *p-tau* phosphorylated tau, *PDD* Parkinson’s disease dementia

*Bold letters indicate an acceptable or higher AUC value (> 0.7)

### Relationships between plasma biomarkers

Spearman’s rank correlation coefficients, along with their respective p values, are summarized in a correlation matrix (Supplementary Fig. 1). Within the PD group, a substantial number of correlations was observed between the interrelationships among the six biomarkers. Conversely, in the HC group, only the relationships between Aβ42 and Aβ40 levels, Aβ42 and Aβ42/40 levels, and NfL and p-tau181 levels showed significant positive correlations.

### Modeling analysis for the ACE-R and MoCA-J

Table 5 presents the results of the generalized linear model analysis of the effects of plasma biomarkers and possible confounders on the ACE-R and MoCA-J scores. Education, GFAP levels, and NfL levels were significantly associated with the ACE-R scores, while GFAP and NfL levels showed a tendency to correlate with the MoCA-J scores, but not significantly.

**Table 5:** The results of the generalized linear models for ACE-R and MoCA-J based on plasma biomarkers and possible confounders in the PD group

Covariate/factor	Estimate	LR chi-square	*p* value	Lower 95%	Upper 95%
ACE-R					
GFAP	− 0.0008	6.7921	**0.0092**	− 0.0013	− 0.0002
NfL	− 0.0016	8.6069	**0.0033**	− 0.0027	− 0.0005
p-tau181	0.0128	0.7994	0.3713	− 0.0153	0.0406
Aβ40	− 0.0001	0.0048	0.9449	− 0.0017	0.0015
Age at examination	− 0.0026	1.6561	0.1981	− 0.0066	0.0014
Education	0.0233	13.6439	**0.0002**	0.0109	0.0356
HY	− 0.0254	3.0001	0.0833	− 0.0542	0.0033
MoCA-J-					
GFAP	− 0.0010	3.4262	0.0642	− 0.0021	0.0001
NfL	− 0.0021	3.7233	0.0537	− 0.0042	< 0.0001
Age at examination	− 0.0046	1.4057	0.2358	− 0.0123	0.0030
Education	0.0115	1.0167	0.3133	− 0.0108	0.0336
HY	− 0.0406	2.0441	0.1528	− 0.0963	0.0151

*ACE-R* Addenbrooke’s Cognitive Examination-Revised, *MoCA-J* Japanese version of the Montreal Cognitive Assessment, *PD* Parkinson's disease, *GFAP* glial fibrillary acidic protein, *NfL* neurofilament light chain, *p-tau* phosphorylated tau, *Aβ* amyloid beta, *HY* Hoehn − Yahr scale, *LR* likelihood ratio

*Bold letters indicate a correlation with a statistically significant difference

## Discussion

In this study, plasma GFAP exhibited a significant correlation with global cognitive function and all of its subdomains, with a notable increase in the PDD group compared with both the HC and PDND groups. Similarly, plasma NfL demonstrated a significant correlation with global cognitive function and exhibited a significant increase in the PDD group when compared with that in the HC group. However, unlike plasma GFAP, plasma NfL also displayed a significant correlation with disease severity and was increased in the PDND group compared with that in the HC group. Although Aβ42/40 and p-tau181 did not show any correlation with global cognitive function, both a significant decrease in Aβ42/40 and an increase in p-tau181 levels in the PDD group compared with those in the PDND group supported prior research indicating the presence of AD pathology in the PDD group. Intriguingly, a distinctive finding was observed for Aβ42/40, with levels increasing in the PDND group compared with those in HCs but decreasing in the PDD group. Taken together, plasma GFAP and NfL levels may reflect widespread reactive astrogliosis or neuronal damage in PD before the onset of AD-related neurodegeneration. The study also indicated that AD pathology may be involved in the development of PDD.

### Plasma GFAP in PD patients

Plasma GFAP serves as a biomarker for astrocytic activation [28], and reactive astrogliosis is increasingly being implicated in PD pathogenesis [29]. Pathological studies of the post mortem brains of patients with Lewy body disorder have revealed that astrocytic alpha-synuclein accumulation contributes significantly to alpha-synuclein pathology [30]. Although the relationship between plasma GFAP levels and clinical scores in PD patients remains incompletely understood, recent studies have demonstrated higher plasma GFAP levels in PD patients with dementia and mild cognitive impairment than in controls [14, 31]. Moreover, these studies identified a significant negative correlation between plasma GFAP levels and MMSE scores in all participants with PD.

Neural networks that cause cognitive symptoms in patients with PDD are widely distributed and diverse, with overlapping functions that depend on primary neurotransmitters [32]. Evidence suggests that damage to one network may influence another, as neurotransmitters can modulate each other’s effects [33], although the cellular-level pathology in PDD is heterogeneous, and the effects of different genes are still being uncovered [32]. Our findings revealed that plasma GFAP levels were negatively correlated with not only the total MMSE, MoCA-J, ACE-R, and FAB scores but also with all subcategories of the MoCA-J and ACE-R. These findings suggest that astrocytic pathology may be a common feature of cognitive decline in PD irrespective of the involvement of different cognitive networks.

### Plasma NfL in PD patients

Plasma NfL is a component of the neuronal cytoskeleton, and increased plasma NfL has been shown to serve as a biomarker for a variety of neurodegenerative diseases [11]. Although controversy remains as to whether plasma NfL levels are elevated in patients with PD compared with those in HCs [14, 34–36], the NfL value has been significantly associated with both motor severity and cognitive decline in PD [37–39], suggesting its usefulness as a disease progression marker in PD. We confirmed that plasma NfL was negatively correlated with MMSE, ACE-R, MoCA-J, and FAB scores, although the correlations tended to be weaker than those of GFAP. Plasma NfL, in contrast to GFAP, was also positively correlated with the MDS-UPDRS Part II and HY scale. GFAP and NfL could be biomarkers of cognitive decline in PD, but it should be considered that NfL can also reflect motor symptoms.

### Plasma p-tau 181 in PD patients

Plasma p-tau181 is a useful diagnostic and prognostic biomarker of AD, correlates with cerebrospinal fluid (CSF) p-tau181, and predicts Aβ and tau positivity on PET [10]. Evidence suggests that tau is involved in the pathophysiology of PD, with tau and alpha-synuclein colocalizing in Lewy bodies [40]. Moreover, a genome-wide association study identified MAPT, the gene encoding the tau protein, as a risk factor for PD [41].

Higher plasma p-tau181 levels have been reported in patients with PD than in HCs, but no significant association has been observed between plasma p-tau181 levels and PD-related clinical indices, including the HY and cognitive scales [13, 35]. In our study, patients with PD showed a tendency toward higher plasma p-tau181 levels than those in HCs, but the difference was not significant. Only patients with PDD showed a significant increase in plasma p-tau181 levels when compared with HCs. Although the clinical significance of plasma p-tau181 in PD remains unclear, our study suggests its potential relevance in advanced cognitive decline in PD.

### Plasma Aβ in PD patients

Previous studies have demonstrated that plasma Aβ42/40 levels decrease in individuals with amyloid PET-positive AD [9, 42]. Although the global Aβ load on PET is negatively associated with memory and language functions [43], the relationship between plasma Aβ42/40 levels and Aβ pathology in the brain has not been fully investigated in PD.

Our study found that plasma Aβ42/40 levels were significantly higher in patients with PD, especially PDND, than those in HCs, which is consistent with a previous report regarding the use of plasma biomarkers for the differential diagnosis of Parkinson syndromes [44]. The detailed mechanisms underlying the higher plasma Aβ42/40 levels in patients with PD remain unknown, but one possible explanation is that alpha-synuclein uptake may interfere with monomeric Aβ40 [45]. Another study showed decreased Aβ40 levels with increased alpha-synuclein levels in patients with PD [46]. A head-to-head comparison study of plasma biomarkers in multiple system atrophy, a synucleinopathy similar to PD, demonstrated decreased Aβ40 and increased Aβ42/40 levels, which supports this hypothesis [47]. Although plasma Aβ40 levels were not significantly decreased in patients with PD compared with those in HCs, alpha-synuclein may influence plasma Aβ levels in patients with PD.

However, multiple comparison results showed decreased plasma Aβ42/40 levels in patients with PDD compared with those in PDND patients but not with those in HCs. ROC analysis also showed that decreased plasma Aβ42/40 levels could be a supportive finding to distinguish patients with PDD from those with PDND. This could suggest that amyloid pathology is more developed in patients with PDD than in patients with PDND which is consistent with a previous meta-analysis demonstrating the involvement of amyloid pathology in the development of PDD [48]. Our study indicated plasma Aβ42/40 levels might exhibit divergent changes in patients with PD without cognitive impairment and in patients with PDD.

Because plasma Aβ40, Aβ42, and Aβ42/40 levels showed limited correlations with plasma GFAP, NfL, and p-tau 181 levels compared with those of AD patients, the dynamics of plasma Aβ42/40 in PD may differ from those of amyloid PET and CSF Aβ42/40. Further studies in larger PD cohorts with PET imaging, CSF, and blood measurements of Aβ and alpha-synuclein pathology are needed to provide more insight.

### Significance of plasma biomarkers in the clinical presentation of PD

The results of this study suggest that in cognitive impairment in PD, elevated plasma GFAP and NfL levels precede the appearance of abnormalities in Aβ 42/40 and p-tau181 levels, which occur only after marked progression of dementia. Pathological analysis of post mortem brains showed an increased severity of alpha-synuclein pathology in the limbic and cortical regions of PDD patients compared with those of PD patients with normal cognition, but no change was observed in the severity of tau or Aβ pathology [49]. Autopsy studies have reported an association between plasma GFAP and AD pathology including Aβ and tau in Lewy body spectrum disorders [50]. In contrast, our results showed that Aβ42/40 levels were decreased in the PDD group compared with those in the PDND group and that p-tau181 levels were increased in the PDD group compared with those in HCs. This finding is consistent with the reports that PD with Aβ accumulation is a significant predictor of cognitive decline [51] and that approximately one-third to half of patients with PDD exhibit abnormal Aβ accumulation on PET [52, 53].

Traditionally, cognitive dysfunction in PD is thought to be preceded by frontal lobe-based deficits in working memory, executive function, and attention [54]. However, the recent investigations suggest that a wide range of domains is impaired, including executive function, memory, visuospatial function, attention, and language [55]. The pathogenesis of this clinical condition is difficult to explain by the alpha-synuclein propagation hypothesis and complications of AD pathology. The failure of numerous clinical trials targeting cognitive decline in PD to date also suggests a need to consider a new pathological hypothesis [56]. Our study showed that the etiology of cognitive decline in PD is intricate, with astrocytic lesions playing a significant role, while the impacts of alpha-synuclein, tau, and Aβ pathology may differ depending on the severity of cognitive decline. AD-related plasma biomarkers could be valuable in elucidating the underlying pathological mechanisms of cognitive decline in PD and in developing preventative measures to mitigate this condition.

## Limitations

First, this was a single-center study, and the number of participants was relatively limited. Second, diagnoses of our participants were based on the clinical evaluations rather than neuropathological confirmation. Third, we did not perform the comparison of AD-related plasma biomarkers to patients with AD and patients with dementia with Lewy bodies. Fourth, AD-related CSF biomarkers including CSF Aβ42/40, CSF p-tau, and CSF NfL for cross comparison were not examined. Finally, we did not conduct PET imaging to detect Aβ and tau; therefore, the neocortical burden of Aβ and tau related to AD pathology was not evaluated. Further studies considering these limitations would be important for verifying our findings and analyses in this study.

## Conclusion

This study examined the association between six plasma biomarkers and cognitive decline in patients with PD. The results demonstrated that plasma GFAP is a reliable indicator of cognitive decline, while NfL captured both cognitive decline and disease severity in both the PDND and PDD groups. Although the plasma Aβ42/40 and p-tau181 levels were not correlated with ACE-R and MoCA-J scores, they showed noteworthy changes in the PDD group, suggesting the involvement of AD pathology in severe cognitive decline in PD patients.

### Supplementary Information

Below is the link to the electronic supplementary material.

Supplementary file1 (PDF 432 KB)

Supplementary file2 (PDF 168 KB)

## Data Availability

The data generated during this study are available from the corresponding author upon reasonable request.

## Abbreviations

Aβ: Amyloid beta
ACE-R: Addenbrooke’s Cognitive Examination-Revised
AUC: Area under the curve
FAB: Frontal Assessment Battery
GFAP: Glial fibrillary acidic protein
HC: Healthy control
HY: Hoehn and Yahr
MDS-UPDRS: Movement Disorder Society’s Unified PD Rating Scale
MMSE: Mini-Mental State Examination
MoCA-J: Japanese version of the Montreal Cognitive Assessment
NfL: Neurofilament light chain
OSIT-J: Odor Stick Identification Test for Japanese
PET: Positron emission tomography
PD: Parkinson’s disease
PDD: Parkinson’s disease dementia
PDND: Nondemented Parkinson’s disease
p-tau: Phosphorylated tau
QOL: Quality of life
ROC: Receiver operating characteristic
Simoa: Single-molecule array

## References

[1] Dorsey ER, Sherer T, Okun MS, Bloemd BR (2018). The emerging evidence of the Parkinson pandemic. J Parkinsons Dis.

[2] Leroi I, McDonald K, Pantula H, Harbishettar V (2012). Cognitive impairment in Parkinson disease: impact on quality of life, disability, and caregiver burden. J Geriatr Psychiatry Neurol.

[3] Severiano E, Sousa C, Alarcão J, Martins IP, Ferreira JJ (2022). Frequency of dementia in Parkinson’s disease: a systematic review and meta-analysis. J Neurol Sci.

[4] Sabbagh MN, Adler CH, Lahti TJ (2009). Parkinson disease with dementia: comparing patients with and without Alzheimer pathology. Alzheimer Dis Assoc Disord.

[5] Hurtig HI, Trojanowski JQ, Galvin J (2000). Alpha-synuclein cortical Lewy bodies correlate with dementia in Parkinson’s disease. Neurology.

[6] Aarsland D, Batzu L, Halliday GM (2021). Parkinson disease-associated cognitive impairment. Nat Rev Dis Primers.

[7] Hansson O (2021). Biomarkers for neurodegenerative diseases. Nat Med.

[8] Pichet Binette A, Janelidze S, Cullen N (2023). Confounding factors of Alzheimer’s disease plasma biomarkers and their impact on clinical performance. Alzheimers Dement.

[9] Schindler SE, Bollinger JG, Ovod V (2019). High-precision plasma β-amyloid 42/40 predicts current and future brain amyloidosis. Neurology.

[10] Janelidze S, Mattsson N, Palmqvist S (2020). Plasma P-tau181 in Alzheimer’s disease: relationship to other biomarkers, differential diagnosis, neuropathology and longitudinal progression to Alzheimer’s dementia. Nat Med.

[11] Ashton NJ, Janelidze S, Al Khleifat A (2021). A multicentre validation study of the diagnostic value of plasma neurofilament light. Nat Commun.

[12] Pereira JB, Janelidze S, Smith R (2021). Plasma GFAP is an early marker of amyloid-β but not tau pathology in Alzheimer’s disease. Brain.

[13] Pagonabarraga J, Pérez-González R, Bejr-kasem H (2022). Dissociable contribution of plasma NfL and p-tau181 to cognitive impairment in Parkinson’s disease. Parkinsonism Relat Disord.

[14] Tang Y, Han L, Li S (2023). Plasma GFAP in Parkinson’s disease with cognitive impairment and its potential to predict conversion to dementia. NPJ Parkinsons Dis.

[15] Litvan I, Goldman JG, Tröster AI (2012). Diagnostic criteria for mild cognitive impairment in Parkinson’s disease: Movement Disorder Society Task Force guidelines. Mov Disord.

[16] Emre M, Aarsland D, Brown R (2007). Clinical diagnostic criteria for dementia associated with Parkinson’s disease. Mov Disord.

[17] Tang Y, Liang X, Han L (2020). Cognitive function and quality of life in Parkinson’s disease: a cross-sectional study. J Parkinsons Dis.

[18] Postuma RB, Berg D, Stern M (2015). MDS clinical diagnostic criteria for Parkinson’s disease. Mov Disord.

[19] Lin YS, Lee WJ, Wang SJ, Fuh JL (2018). Levels of plasma neurofilament light chain and cognitive function in patients with Alzheimer or Parkinson disease. Sci Rep.

[20] Dubois B, Burn D, Goetz C (2007). Diagnostic procedures for Parkinson’s disease dementia: recommendations from the Movement Disorder Society Task Force. Mov Disord.

[21] dos Santos Kawata KH, Hashimoto R, Nishio Y (2012). A validation study of the Japanese Version of the Addenbrooke’s Cognitive Examination-Revised. Dement Geriatr Cogn Dis Extra.

[22] Mioshi E, Dawson K, Mitchell J (2006). The Addenbrooke’s Cognitive Examination Revised (ACE-R): a brief cognitive test battery for dementia screening. Int J Geriatr Psychiatry.

[23] Goetz CG, Tilley BC, Shaftman SR (2008). Movement Disorder Society-Sponsored Revision of the Unified Parkinson’s Disease Rating Scale (MDS-UPDRS): scale presentation and clinimetric testing results. Mov Disord.

[24] Dubois B, Slachevsky A, Litvan I, Pillon B (2000). The FAB: a frontal assessment battery at bedside. Neurology.

[25] Nasreddine ZS, Phillips NA, Bédirian V (2005). The Montreal Cognitive Assessment, MoCA: a brief screening tool for mild cognitive impairment. J Am Geriatr Soc.

[26] Tomlinson CL, Stowe R, Patel S (2010). Systematic review of levodopa dose equivalency reporting in Parkinson’s disease. Mov Disord.

[27] Schade S, Mollenhauer B, Trenkwalder C (2020). Levodopa equivalent dose conversion factors: an updated proposal including Opicapone and safinamide. Mov Disord Clin Pract.

[28] Yang Z, Wang KKW (2015). Glial fibrillary acidic protein: from intermediate filament assembly and gliosis to neurobiomarker. Trends Neurosci.

[29] Rizor A, Pajarillo E, Johnson J (2019). Astrocytic oxidative/nitrosative stress contributes to parkinson’s disease pathogenesis: the dual role of reactive astrocytes. Antioxidants.

[30] Altay MF, Liu AKL, Holton JL (2022). Prominent astrocytic alpha-synuclein pathology with unique post-translational modification signatures unveiled across Lewy body disorders. Acta Neuropathol Commun.

[31] Oeckl P, Halbgebauer S, Anderl-Straub S (2019). Glial fibrillary acidic protein in serum is increased in Alzheimer’s disease and correlates with cognitive impairment. J Alzheimers Dis.

[32] Gratwicke J, Jahanshahi M, Foltynie T (2015). Parkinson’s disease dementia: a neural networks perspective. Brain.

[33] Calabresi P, Picconi B, Parnetti L, Di FM (2006). Personal View A convergent model for cognitive dysfunctions in Parkinson’s disease: the critical dopamine-acetylcholine synaptic balance. Lancet Neurol.

[34] Marques TM, Van Rumund A, Oeckl P (2019). Serum NFL discriminates Parkinson disease from atypical parkinsonisms. Neurology.

[35] Batzu L, Rota S, Hye A (2022). Plasma p-tau181, neurofilament light chain and association with cognition in Parkinson’s disease. NPJ Parkinsons Dis.

[36] Wong YY, Wu CY, Yu D (2022). Biofluid markers of blood-brain barrier disruption and neurodegeneration in Lewy body spectrum diseases: a systematic review and meta-analysis. Parkinsonism Relat Disord.

[37] Aamodt WW, Waligorska T, Shen J (2021). Neurofilament light chain as a biomarker for cognitive decline in Parkinson disease. Mov Disord.

[38] Zhu Y, Yang B, Wang F (2021). Association between plasma neurofilament light chain levels and cognitive function in patients with Parkinson’s disease. J Neuroimmunol.

[39] Su Lyn Ng A, Jayne Tan Y, Cui Wen Yong A (2020). Utility of plasma Neurofilament light as a diagnostic and prognostic biomarker of the postural instability gait disorder motor subtype in early Parkinson’s disease. Mol Neurodegener.

[40] Arima K, Hirai S, Sunohara N (1999). Cellular co-localization of phosphorylated tau-and NACPra-synuclein-epitopes in Lewy bodies in sporadic Parkinson’s disease and in dementia with Lewy bodies. Brain Res.

[41] Chang D, Nalls MA, Hallgrímsdóttir IB (2017). A meta-analysis of genome-wide association studies identifies 17 new Parkinson’s disease risk loci. Nat Genet.

[42] Nakamura A, Kaneko N, Villemagne VL (2018). High performance plasma amyloid-β biomarkers for Alzheimer’s disease. Nature.

[43] Baik K, Kim HR, Park M (2023). Effect of amyloid on cognitive performance in Parkinson’s disease and dementia with Lewy bodies. Mov Disord.

[44] Li Q, Li Z, Han X (2022). A panel of plasma biomarkers for differential diagnosis of parkinsonian syndromes. Front Neurosci.

[45] Chan DKY, Braidy N, Xu YH (2016). Interference of α-synuclein uptake by monomeric β-amyloid1-40 and potential core acting site of the interference. Neurotox Res.

[46] Chen NC, Chen HL, Li SH (2020). Plasma levels of α-synuclein, aβ-40 and t-tau as biomarkers to predict cognitive impairment in Parkinson’s disease. Front Aging Neurosci.

[47] Guo Y, Shen XN, Huang SY (2023). Head-to-head comparison of 6 plasma biomarkers in early multiple system atrophy. NPJ Parkinsons Dis.

[48] Hu X, Yang Y, Gong D (2017). Changes of cerebrospinal fluid Aβ42, t-tau, and p-tau in Parkinson’s disease patients with cognitive impairment relative to those with normal cognition: a meta-analysis. Neurol Sci.

[49] Kouli A, Camacho M, Allinson K, Williams-Gray CH (2020). Neuroinflammation and protein pathology in Parkinson’s disease dementia. Acta Neuropathol Commun.

[50] Cousins KAQ, Irwin DJ, Chen-Plotkin A (2023). Plasma GFAP associates with secondary Alzheimer’s pathology in Lewy body disease. Ann Clin Transl Neurol.

[51] Myers PS, O’Donnell JL, Jackson JJ (2022). Proteinopathy and longitudinal cognitive decline in Parkinson disease. Neurology.

[52] Petrou M, Dwamena BA, Foerster BR (2015). Amyloid deposition in Parkinson’s disease and cognitive impairment: a systematic review. Mov Disord.

[53] Palermo G, Tommasini L, Aghakhanyan G (2019). Clinical correlates of cerebral amyloid deposition in Parkinson’s disease dementia: evidence from a PET study. J Alzheimers Dis.

[54] Kehagia AA, Robbins FRSTW, Robbins TW (2010). Neuropsychological and clinical heterogeneity of cognitive impairment and dementia in patients with Parkinson’s disease. Lancet Neurol.

[55] Carceles-Cordon M, Weintraub D, Chen-Plotkin AS (2023). Cognitive heterogeneity in Parkinson’s disease: a mechanistic view. Neuron.

[56] Bayram E, Batzu L, Tilley B (2023). Clinical trials for cognition in Parkinson’s disease: Where are we and how can we do better?. Parkinsonism Relat Disord.

