# Supplementary material for: Associations of Alzheimer's-related plasma biomarkers with cognitive decline in Parkinson's disease
Source: J Neurol. 2023 Jul 22;270(11):5461–74. doi: 10.1007/s00415-023-11875-z (PMC10576723; doi:10.1007/s00415-023-11875-z)
Supplement: Supplementary file 1 — Supplementary file1 (PDF 432 KB) [file 415_2023_11875_MOESM1_ESM.pdf]

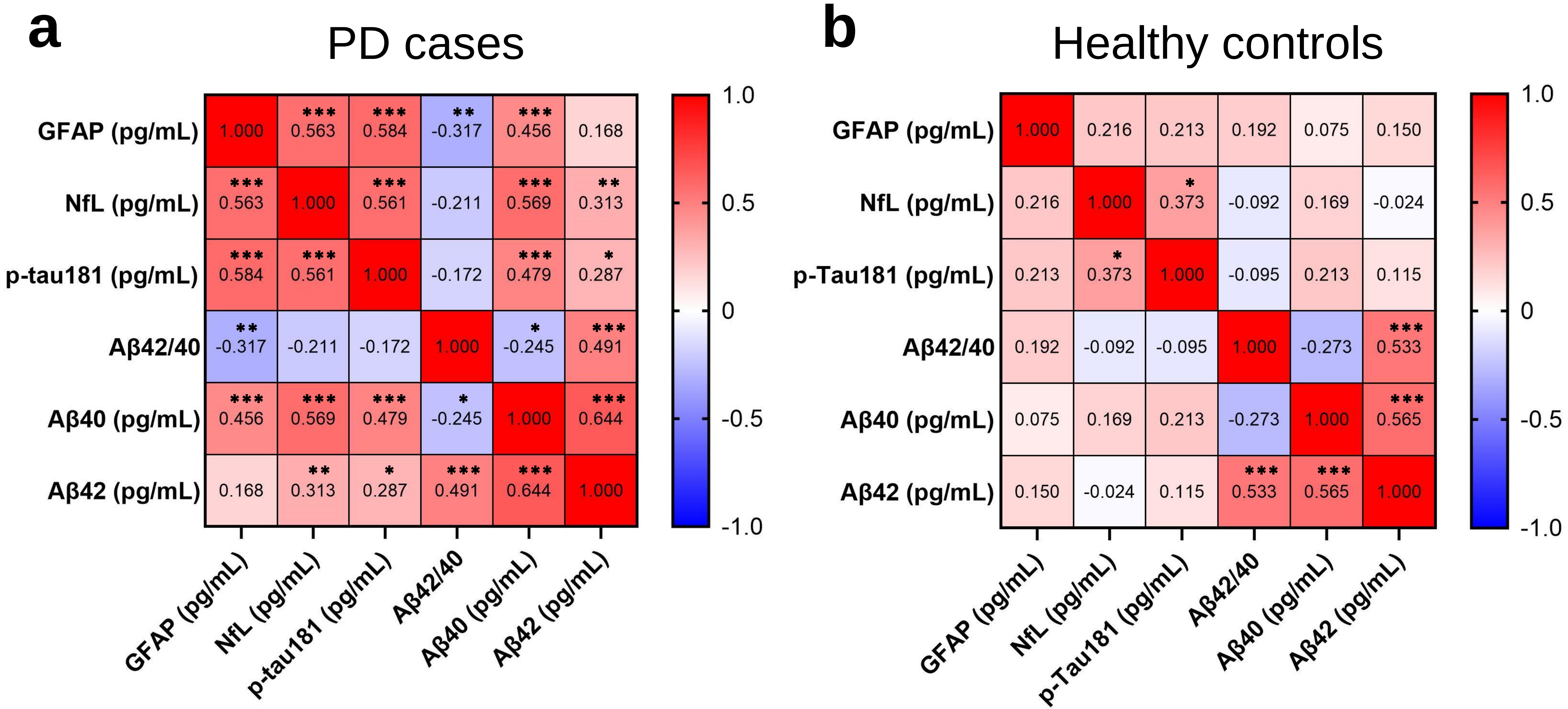

Supplementary Fig. 1. Spearman's rank correlation between plasma biomarkers in each group. **a** Spearman's rank correlation between plasma biomarkers in the PD group. **b** Spearman's rank correlation between plasma biomarkers in the HC group. Spearman's rank correlation test was used to determine significant correlations. PD, Parkinson's disease; HC, healthy control; GFAP, glial fibrillary acidic protein; NfL, neurofilament light chain; p-tau, phosphorylated tau; Aβ, amyloid beta. * $p < 0.05$; ** $p < 0.01$; *** $p < 0.001$.
