# Supplementary material for: Associations of Alzheimer's-related plasma biomarkers with cognitive decline in Parkinson's disease
Source: J Neurol. 2023 Jul 22;270(11):5461–74. doi: 10.1007/s00415-023-11875-z (PMC10576723; doi:10.1007/s00415-023-11875-z)
Supplement: Supplementary file 2 — Supplementary file2 (PDF 168 KB) [file 415_2023_11875_MOESM2_ESM.pdf]

Supplementary Table 1.

The clinical characteristics and levels of plasma biomarkers of the participants in the HC, PDND and PDD group.

Characteristics	HC (N=38)<br>mean ± SD	PDND (N=49)<br>mean ± SD	PDD (N=21)<br>mean ± SD	HC vs. PDND	HC vs. PDD	PDND vs. PDD
**Age at examination (years)** [a]	68.4 ± 4.90	68.0 ± 8.48	73.9 ± 6.74	0.9516	**0.0016**	**0.0104**
**Sex (Male / female)** [b]	19/19	28/21	28/21	0.5241	0.4243	0.7948
**Age at onset (years)** [c]	-	60.0 ± 10.3	67.1 ± 6.28	-	-	**0.0127**
**Disease duration (months)** [c]	-	95.7 ± 51.1	80.8 ± 49.0	-	-	0.2000
**Education (years)** [a]	13.9 ± 1.80	13.3 ± 2.24	11.4 ± 2.89	0.3765	**0.0013**	**0.0121**
**LEDD (mg)** [c]	-	635 ± 335	673 ± 355	-	-	0.7680
**MDS-UPDRS I** [c]	-	10.0 ± 5.65	12.6 ± 6.01	-	-	0.0959
**MDS-UPDRS II** [c]	-	14.7 ± 8.03	16.1 ± 9.20	-	-	0.6524
**MDS-UPDRS III** [c]	-	34.6 ± 16.2	38.1 ± 16.8	-	-	0.3997
**MDS-UPDRS IV** [c]	-	5.53 ± 4.29	5.11 ± 5.01	-	-	0.6934
**HY** [c]	-	2.96 ± 0.999	3.48 ± 1.08	-	-	**0.0372**
**PDQ-39 SI** [c]	-	28.7 ± 16.1	33.3 ± 14.9	-	-	0.2676

**SCOPA-AUTO** [c]	-	14.9 ± 7.93	15.3 ± 8.46	-	-	0.8172
**GDS-15** [a]	2.55 ± 2.16	6.27 ± 3.27	8.43 ± 4.34	**< 0.0001**	**< 0.0001**	0.0932
**J-QUIP** [c]	-	0.653 ± 0.969	0.667 ± 1.02	-	-	0.9476
**RBDSQ-J** [c]	-	4.88 ± 2.85	6.10 ± 3.06	-	-	0.0946
**ESS** [c]	-	9.24 ± 6.32	9.10 ± 4.82	-	-	0.8021
**OSIT-J score** [c]	-	4.29 ± 2.53	1.90 ± 1.62	-	-	**0.0003**
**MMSE** [a]	28.7 ± 1.19	27.9 ± 1.80	21.4 ± 5.15	0.1190	**< 0.0001**	**< 0.0001**
**ACE-R total score** [a]	95.0 ± 2.99	89.3 ± 8.88	68.3 ± 17.4	**0.0045**	**< 0.0001**	**< 0.0001**
**MoCA-J total score** [a]	24.8 ± 2.67	23.0 ± 3.30	16.6 ± 5.85	**0.0234**	**< 0.0001**	**< 0.0001**
**FAB** [c]	**-**	13.7 ± 2.51	11.4 ± 2.91	-	-	**0.0020**
**GFAP (pg/mL)** [a]	115 ± 39.1	127 ± 60.0	185 ± 60.7	0.8870	**< 0.0001**	**0.0008**
**NfL (pg/mL)** [a]	13.9 ± 4.45	31.1 ± 21.3	59.7 ± 44.0	**< 0.0001**	**< 0.0001**	**0.0291**
**p-tau181 (pg/mL)** [a]	1.62 ± 0.684	2.02 ± 1.28	2.46 ± 1.45	0.6331	**0.0472**	0.2616
**Aβ42/40** [a]	0.0646 ± 0.0139	0.0733 ± 0.0119	0.0653 ± 0.0133	**0.0001**	0.9266	**0.0195**
**Aβ40 (pg/mL)** [a]	100 ± 15.8	92.0 ± 17.0	105 ± 23.9	0.1449	0.8235	0.1155

**Aβ42 (pg/mL)** [a]	6.43 ± 1.49	6.71 ± 1.60	6.78 ± 1.85	0.7599	0.7131	0.9450

HC, healthy control; PDND, non-demented Parkinson's disease; PDD, Parkinson's disease dementia: LEDD, Levodopa equivalent daily dose; MDS-UPDRS, Movement Disorder Society's Unified Parkinson's Disease Rating Scale; HY, Hoehn-Yahr scale; PDQ-39 SI, Parkinson's Disease Questionnaire-39 Summary Index; SCOPA-AUT, Scales for Outcomes in Parkinson's Disease-Autonomic; GDS-15, Geriatric Depression Scale-15; J-QUIP, Japanese version of the Questionnaire for Impulsive-Compulsive Disorders in Parkinson's Disease; RBDSQ-J, Japanese version of the REM Sleep Behavior Disorder Screening Questionnaire; ESS, Epworth Sleepiness Scale; OSIT-J, Odor Stick Identification Test for Japanese; MMSE, Mini-Mental State Examination; ACE-R, Addenbrooke's Cognitive Examination-Revised; MoCA-J, Japanese version of the Montreal Cognitive Assessment; FAB, Frontal Assessment Battery; GFAP, glial fibrillar acidic protein; NfL, neurofilament light chain; p-tau, phosphorylated tau; Aβ, amyloid beta.

[a] Kruskal-Wallis test followed by post hoc Steel-Dwass multiple comparison tests.

[b] Fisher's exact test.

[c] Wilcoxon rank sum test.

*Bold letters indicate a statistically significant difference.
